# Stabilizing leaning postures with feedback controlled functional neuromuscular stimulation after trunk paralysis

**DOI:** 10.3389/fresc.2023.1222174

**Published:** 2023-09-28

**Authors:** Aidan R. W. Friederich, Lisa M. Lombardo, Kevin M. Foglyano, Musa L. Audu, Ronald J. Triolo

**Affiliations:** ^1^ Department of Biomedical Engineering, Case Western Reserve University, Cleveland, OH, United States; ^2^ Advanced Platform Technology Center, Louis Stokes Cleveland Veterans Affairs Medical Center, Cleveland, OH, United States

**Keywords:** spinal cord injury, functional neuromuscular stimulation, seated balance, feedback control, muscle synergies

## Abstract

Spinal cord injury (SCI) can cause paralysis of trunk and hip musculature that negatively impacts seated balance and ability to lean away from an upright posture and interact fully with the environment. Constant levels of electrical stimulation of peripheral nerves can activate typically paralyzed muscles and aid in maintaining a single upright seated posture. However, in the absence of a feedback controller, such seated postures and leaning motions are inherently unstable and unable to respond to perturbations. Three individuals with motor complete SCI who had previously received a neuroprosthesis capable of activating the hip and trunk musculature volunteered for this study. Subject-specific muscle synergies were identified through system identification of the lumbar moments produced via neural stimulation. Synergy-based calculations determined the real-time stimulation parameters required to assume leaning postures. When combined with a proportional, integral, derivative (PID) feedback controller and an accelerometer to infer trunk orientation, all individuals were able to assume non-erect postures of 30–40° flexion and 15° lateral bending. Leaning postures increased forward reaching capabilities by 10.2, 46.7, and 16 cm respectively for each subject when compared with no stimulation. Additionally, the leaning controllers were able to resist perturbations of up to 90 N, and all subjects perceived the leaning postures as moderately to very stable. Implementation of leaning controllers for neuroprostheses have the potential of expanding workspaces, increasing independence, and facilitating activities of daily living for individuals with paralysis.

## 1. Introduction

A major effect of spinal cord injury (SCI) at and above low thoracic levels is reduced ability to maintain seated postures. The need for improved seated stability has been repeatedly identified as an important target for recovery (1–3). A stable trunk provides the necessary base of support for all seated activities as it is part of the biomechanical chain for reaching with the upper extremities (4) and for movements of the head (5). Reaching objects, even within arm length, involves movement of the trunk towards the target (6). However, individuals with SCI compensate for a lack of trunk control during reaching by leaning their trunk in the opposite direction to counterbalance the moment generated by the weight of their outstretched arm and the desired object (7). For objects out of reach, individuals with SCI rely on one arm for support making manipulation of distant objects with two hands almost impossible.

Application of constant levels of functional neuromuscular stimulation (FNS) to activate hip and trunk muscles can increase reaching distances and improve seated posture (8–10). Incorporating feedback control has maintained seated postures in the presence of perturbations (11, 12) and has quickened return to upright motions after reaching objects (13). However, FNS systems have focused primarily on maintaining upright postures without the ability to restore seated leaning, an important aspect of many activities of daily living. Restoring leaning postures would provide greater options for users of neuroprostheses to manipulate objects, exert control over their environments, and increase their available workspace. In this manuscript we describe the development and verification of a non-erect posture stabilizing controller to maintain leaning postures in individuals with SCI using FNS.

Control of neuromuscular systems is complicated because the number of degrees of freedom greatly exceed that required to accomplish a task (the degree of freedom problem). Additionally, each degree of freedom is affected by multiple actuators that work in synchrony to accomplish a movement (the muscle redundancy problem). Methods of addressing these problems have been most studied in FNS control of the arm for reaching. Wolf et al. (14) demonstrated the ability for an individual with complete arm paralysis to maintain static arm positions with FNS and later to follow a trajectory (15). This was accomplished by placing the arm in a Haptic Master and mapping the forces necessary to hold the arm at a fixed location, as well as the forces produced from stimulation. Once these maps were obtained, real-time quasi-Newton optimization was performed to determine the required stimulation parameters for nine stimulus channels to produce the required forces at the wrist. Alternatively, Razavian et al. (16) leveraged muscle synergies to reduce dimensionality and simplify a similar controller. Muscle synergies or modules have been proposed as a possible mechanism for the nervous system to reduce the number of control signals required to perform movements (17, 18). These synergies have been identified in able-bodied individuals (19–21) and even observed to be preserved in some form after incomplete SCI (22, 23). With the synergy method, Razavian et al. reduced six stimulation channels to four synergies, which was able to control reaching in two dimensions in able-bodied individuals.

The goal of our work is to design and deploy feedback control systems that would stabilize seated leaning postures for individuals with paralyzed torso and pelvic musculature. We targeted control of two main degrees of freedom of the hip and lumbar spine: trunk pitch (flexion and extension of the lumbar and hip joint combined) and trunk roll (lateral bending at the lumbar spine). Trunk axial rotation was not considered at this time as it is not critical for leaning. A proportional, integral, derivative (PID) controller was employed in this study because of its previous success in controlling seated posture (11, 12) and because the extrinsic (active muscles) properties of the trunk are dominated by the proportional and derivative model components (24). The integral component was added to help compensate for any steady state errors. The synergy-based control architecture was leveraged to address muscle redundancy and enable real-time optimization of the required stimulation parameters. Such a controller would be required to hold various leaning postures and allow users to extend their reaching abilities while also resisting potentially destabilizing perturbations. To address these needs, we formulated and tested the following hypothesis: A leaning feedback controller will enable perturbation resistant leaning postures that are perceived as stable, while expanding available workspace compared to no stimulation and constant stimulation.

## 2. Methods

### 2.1. Overview

**Figure 1:**
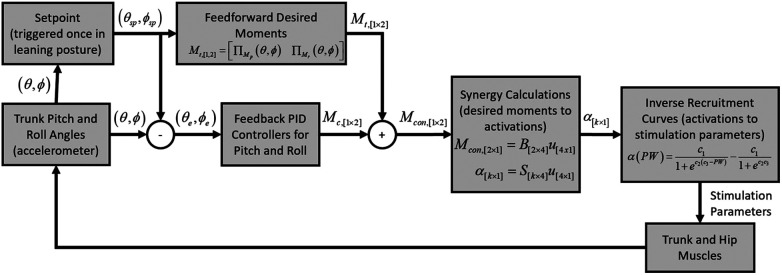
Controller block diagram. Trunk pitch (θ) and roll (ϕ) angles were determined by an accelerometer. Setpoint error $\left(\theta_{e},\phi_{e}\right)$ was obtained by subtracting the setpoint posture $\left(\theta_{sp},\phi_{sp}\right)$ and served as the input to two PID controllers. The target moments $\left(M_{t}\right)$ were determined from plane fits (Π) and added to the moment output of the controllers $\left(M_{\mathrm{con}}\right)$ to yield the resulting moment command. Activation coefficients (α) were calculated through a synergy method and converted to stimulation parameters with reverse recruitment curves.

The system for stabilizing leaning postures was composed of a sensor to determine trunk orientation, a feedback controller, a feedforward controller, a synergy-based optimization, recruitment curves, and an implanted neuroprosthesis (Figure 1). Each of these components are described in detail below.

### 2.2. Participants and neuroprosthesis

Three individuals with SCI participated in this study. Subject neurological and anthropometric characteristics are presented in Table 1. Each participant had previously received an implanted neuroprosthesis for other studies intended to restore standing, walking, or postural balance. The neuroprosthesis is composed of a stimulator-telemeter (25, 26) connected to intramuscular or epimysial electrodes surgically placed to activate nerves serving the muscle groups spanning the trunk and hips. Each subject completed reconditioning exercises at home with the system for several months before the experiments. Additional surface stimulation was applied bilaterally to the quadratus lumborum and erector spinae for subjects S1 and S2. Technical difficulties arising from a malfunctioning chip in the external control unit prevented application of surface stimulation to S3 during the experimental session. All stimulation was applied at an interpulse interval of 50 ms and consisted of charge-balanced asymmetrical cathodic waveforms with the capsule of the implanted pulse generator serving as a common ground. All stimulation was pulse width (PW) modulated with values ranging from 0 to 255 µs. For implanted electrodes, pulse amplitudes were kept constant and ranged from 1.5 mA to 20 mA (see Table 2 for specific values). For surface electrodes, pulse amplitudes were kept constant at 100 mA. Before the experiments, the maximum comfortable stimulation parameters were determined for each channel by slowly increasing stimulation parameters (in PW steps of 10 μs) until hardware limits were reached or subjects reported discomfort. Participants were informed of all aspects of the experiment and subsequently signed consent forms approved by the local institutional review board (IRB: VA Northeast Ohio Healthcare System, Protocol Number: 1591730, Approval Date: 7/2/2021).

**Table 1:** Clinical characteristics of study participants.

Subject	Age (year)	Gender	Height (cm)	Weight (kg)	Injury level	AIS grade^a^	Time post injury (year)	Activated muscle groups
S1	46	F	173	79	T4	A	10	RPA, RGX, RES, RIL, LQL, LIL, LPA, LGX, LES, SRES, SLES, SRQL, SLQL
S2	50	F	168	58.5	C7	B	24	RQL, RGM, RPA, RHS, LPA, RGX, LQL, LGM, RES, RGX2, LES, LGX, LHS, SRES, SLES, SRQL, SLQL
S3	44	M	183	91	C7	B	20	RES, RGX, LES, LQL, LGX, LHS, RQL, RGX, RHS, RPA

Acronyms for muscle targets: R/l, Right/Left; ES, lumbar erector spinae; QL, quadratus lumborum; PA, posterior portion of adductor magnus; GX, gluteus maximus; GM, gluteus medius; IL, iliopsoas; HS, hamstrings (semimembranosus). S indicates surface stimulation.

^a^ American Spinal Injury Association Impairment Score (AIS).

**Table 2:** Stimulation parameters for the constant stimulation condition. Stimulation frequency was 20 Hz.

Channel name	Subject					
	S1		S2		S3	
	PW (μs)	Amp (mA)	PW (μs)	Amp (mA)	PW (μs)	Amp (mA)
Right erector spinae	201	20	81	20	173	20
Right quadratus lumborum	19	12.5	35	20	51	20
Right quadriceps			35	1.5		
Right iliopsoas			248	20		
Right semimembranosus					4	20
Right posterior adductor magnus	37	20	245	20	16	20
Right gluteus maximus	206	20			38	20
Right gluteus maximus 2	193	12.5				
Left erector spinae	223	20	106	20	176	20
Left quadratus lumborum	19	20	145	20		
Left quadriceps			45	1.5		
Left iliopsoas			247	20		
Left semimembranosus					8	20
Left posterior adductor magnus	24	20	248	20		
Left gluteus maximus	32	20			17	20

### 2.3. Feedforward system identification

#### 2.3.1. Trunk moment transducer

**Figure 2:**
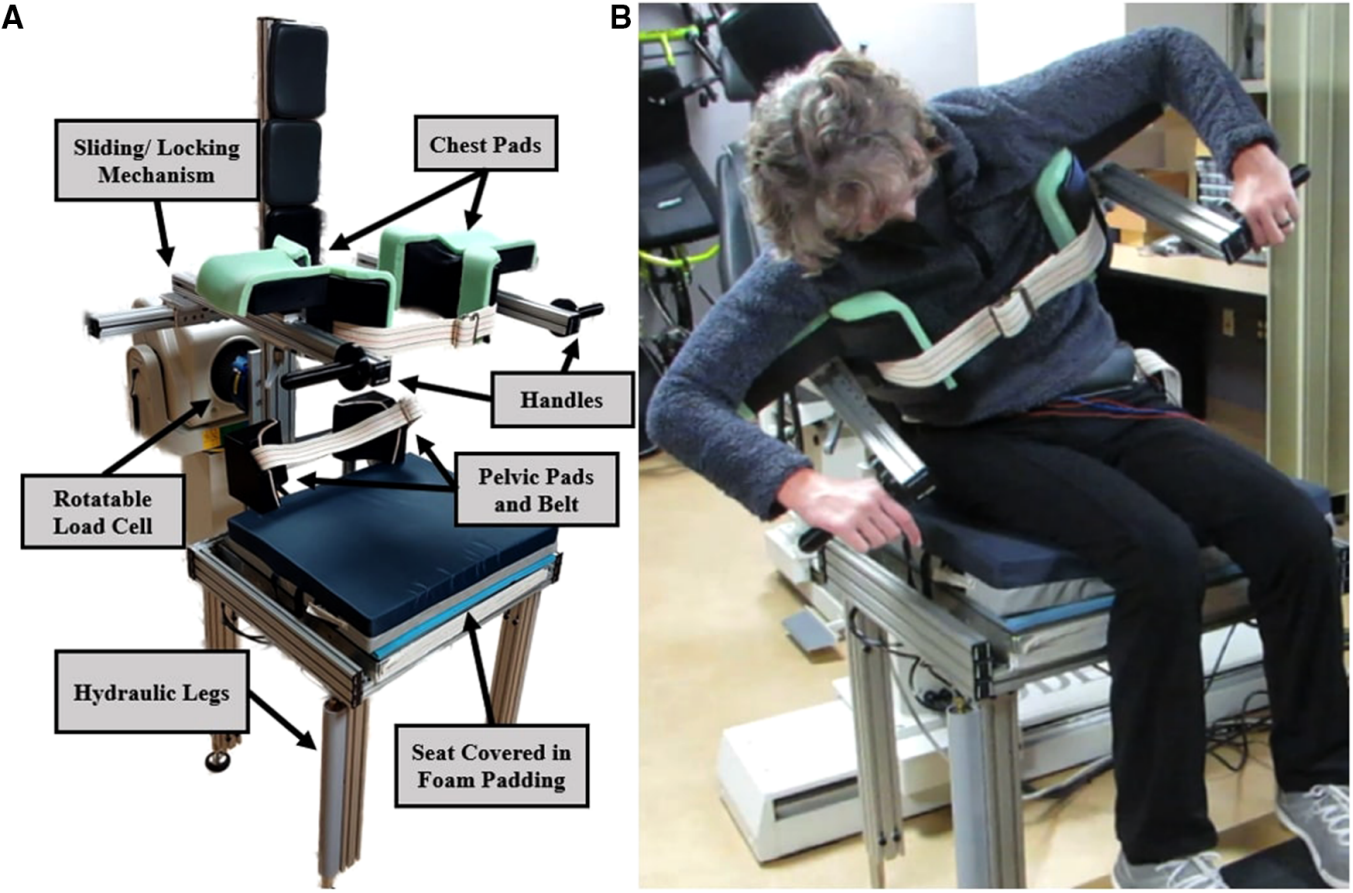
The trunk moment transducer without (**A**) and with (**B**) a subject leaning forward and laterally. Chair height is adjusted until the load cell is in line with the S1 vertebrae. Chest pads are moved into place under the axilla with the sliding/locking mechanism. Pelvic pads ensure proper alignment of the spine during system identification.

A device to record forces and moments from activation of the trunk muscles in multiple dimensions was modified from a previous study (27) to measure moments about the S1 lumbar vertebrae with subjects in various leaning postures (Figure 2). The subject sat on a padded seat and the seat height was adjusted until the S1 vertebrae was aligned with a load cell (65E20A4-I100-EF-250l, JR3 Inc., Woodland, CA) behind the subject. The load cell and device were mounted on a Biodex System 4 Pro (Biodex Medical Systems, Inc, Shirley, New York) to take advantage of the rotational capabilities for different postures. Once seated and at the proper height, pelvic pads were pressed against each side of the hip to align the spine and load cell in the frontal plane. Then the subject assumed an upright posture with the help of an experimenter, and chest pads on sliding rails were positioned under the axillae to stabilize the torso. Once aligned, the sliding mechanisms were locked in place and any moments about the S1 lumbar vertebrae were transmitted to the load cell. Before any stimulation was applied, the subjects were asked to not intervene, and to minimize movements of their upper extremities by holding the support handles provided. Moments in the pitch and roll directions were sampled at 1,000 Hz with a custom Simulink model (Mathworks, Natick, MA) running on a Speedgoat real-time computer (Performance real-time target machine, Speedgoat, Switzerland). All moments obtained from the load cell were filtered offline with a 4th order zero-phase Butterworth filter with a low-pass cutoff of 10 Hz (28).

#### 2.3.2. Recruitment curves

Recruitment curves were obtained with the deconvolved ramp method (14, 29, 30). With the subject in an upright posture, one second of stimulation was applied to the target channel to prime the muscle and avoid possible potentiation effects. To obtain the impulse response of the muscle, we applied a single stimulation pulse four times with two seconds between each pulse. The time from application of stimulation to maximum muscle moment was recorded for each pulse and averaged together for later use in the deconvolution process. Two seconds after the last stimulation burst, we applied a two second stimulation ramp up to the maximum comfortable pulse duration, followed immediately by a two second ramp down to zero. The ramp was repeated four times with two seconds of rest between each ramp. The ramp response was then isolated and deconvolved with a second-order, critically-damped, linear system with system poles determined by the average impulse response time obtained earlier. This was performed for each channel thought to be relevant to seated posture and surface channels bilaterally targeting the lumbar erector spinae and quadratus lumborum for subjects S1 and S2. Channels that did not visually evoke a response above the noise of the load cell were removed from consideration and marked for constant baseline stimulation during the leaning experiment. Recruitment curves were then fit to a sigmoid curve shown in Equation 1 (27), where *α* is the activation coefficient, PW is the stimulation pulse width, and *c*_1_, *c*_2_, *c*_3_ are constants. Constants were determined with a least-squares fitting method.

(1) $$\alpha (\mathrm{PW})=\frac{c_{1}}{1+e^{c_{2}(c_{3}-PW)}}-\frac{c_{1}}{1+e^{c_{2}c_{3}}}.$$

#### 2.3.3. Surface fits of supporting and applied moments

Once recruitment curves were computed, we collected the maximum moments that each muscle could produce at various leaning postures. We applied maximum comfortable stimulation to every channel that visually evoked a moment response while collecting recruitment curves for two seconds with two seconds of rest between delivering stimulation via each channel. This was repeated three times. The stimulation pattern was applied at 28 different postures covering all possible combinations of the following angles: 0°, 10°, 20°, 30° pitch and −30°, −20°, −10°, 0°, 10°, 20°, 30° roll. Adjusting the roll angles was done by rotating the Biodex head and did not require readjustment of the chest pads. Adjusting the pitch angle required removal of the chest pads, adjusting the subject into the correct posture, tilting the Biodex to match the same angle, and replacing the chest pads. A 3-axis accelerometer (Trigno Avanti, Delsys Inc., Natick, MA) was placed at T4 on the subject's back to approximate trunk tilt. Accelerometer signals were recorded at 100 Hz. Accelerometer and load cell data were filtered offline with a 4th order zero-phase Butterworth filter with a low-pass cutoff of 10 Hz (27, 31).

The moments from each stimulation pulse train were visually inspected and removed if they contained movement noise uncorrelated with the application of stimulation. The remaining moment data were processed to determine the moment produced by the muscle in the pitch and roll directions. Pitch and roll moments were defined as moments around the *x* and *z* axis of the load cell, respectively. These correspond to the mediolateral and anterior/posterior axes of the body. Moments about the y-axis were not considered as these correspond to axial rotation. Moments were averaged over one second beginning 0.5 s after stimulation onset. Additionally, baseline moments were averaged over one second beginning 1.5 s before stimulation was applied and subtracted from the average moment during stimulation to determine moments due to stimulation. The baseline moments were also noted as the required supporting moments for that posture. Trunk pitch and roll angles were determined from the accelerometer's tilt and averaged over one second beginning 1.5 s before each stimulation pulse train.

The pitch moments for each channel were plotted against the pitch (θ) and roll (ϕ) angles and fit to a second order polynomial surface P(θ,ϕ). This was repeated for the roll moments as well. The required supporting moments for both pitch and roll directions obtained before each application of stimulation were fit to a plane ∏(θ,ϕ). Root mean squared error (RMSE) and coefficient of determination (*r*^2^) were determined for each fit.

### 2.4. Synergy calculations

The data required to determine the synergies were obtained through a series of optimizations. The system can be modeled with Equation 2.

(2) $$M_{t,[1\times 2]}=\alpha_{\left[1\times k\right]}M_{m,[k\times 2]}$$

$M_{t}$ describes the target moments required to hold a static leaning posture in both the pitch and roll directions. α are the activation coefficients of *k* channels. $M_{m}$ defines the moments produced from maximum stimulation of each channel. Each row represents a different channel. The first column is the resulting pitch moment, and the second column is the resulting roll moment. Equation 2 can be expanded to

(3) $$\left[\begin{array}{cc} \prod_{M_{p}}(\theta ,\phi ) & \prod_{M_{r}}(\theta ,\phi ) \end{array}\right]=\left[\begin{array}{cccc} \alpha_{1} & \alpha_{2} & \cdots & \alpha_{k} \end{array}\right]\left[\begin{array}{cc} P_{M_{p},1}(\theta ,\phi ) & P_{M_{r},1}(\theta ,\phi )\\ P_{M_{p},2}(\theta ,\phi ) & P_{M_{r},2}(\theta ,\phi )\\ \vdots & \vdots \\ P_{M_{p},k}(\theta ,\phi ) & P_{M_{r},k}(\theta ,\phi ) \end{array}\right]$$

where $M_{t}$ was populated with the static posture plane (∏(θ,ϕ)) fits for pitch $\left(M_{p}\right)$ and roll $\left(M_{r}\right)$ moments obtained in section 2.3.3 from the moments required to hold the 28 postures. $M_{m}$ was populated with the second order polynomial surfaces (P(θ,ϕ)) for pitch $\left(M_{p}\right)$ and roll $\left(M_{r}\right)$ moments. Equation 3 was populated at postures every 2° between 0° to 30° pitch angle and −30° to 30° roll angle for a total of 496 different postures. The activation coefficients at each posture were then solved for with the following optimization.

(4) $$\begin{array}{rl} & \underset{\alpha }{\min}\|\alpha_{\left[1\times k\right]}{\|}_{2}\\ & \mathrm{subject}\;\mathrm{to}M_{t,[1\times 2]}=\alpha_{\left[1\times k\right]}M_{m,[k\times 2]}\\ & \;\;\;\;\alpha_{i}\in [0,1]\;\forall i\in [1,2,\ldots ,k] \end{array}$$

We minimized the norm of the activation coefficients with lower and upper bounds of 0 and 1. Cases where a solution could not be found indicated a posture that the individual would be unable to assume with the neuroprosthesis and were not included in later steps. The activation coefficients and targeted moments from every successful optimization (*n* = 496 possible postures—number of postures with no solution, S1: *n* = 347, S2: *n* = 277, S3: *n* = 136) were stored in matrix *A* (Equation 5) and $M_{\mathrm{store}}$ (Equation 6) for determining the synergies.

(5) $$A_{\left[k\times n\right]}=\left[\begin{array}{cccc} {\alpha^{\prime }}_{1} & {\alpha^{\prime }}_{2} & \cdots & {\alpha^{\prime }}_{n} \end{array}\right]$$

(6) $$M_{\mathrm{store},[2\times n]}=\left[\begin{array}{cccc} M_{t,1} & M_{t,2} & \cdots & M_{t,n} \end{array}\right]$$

The synergies were identified by applying Non-Negative Matrix Factorization (NNMF) to Equation 7.

(7) $$A_{\left[k\times n\right]}≃S_{\left[k\times 4\right]}C_{\left[4\times n\right]}$$

NNMF is a common method for determining synergies (16, 17, 21, 32). We decided on identifying four synergies as it is the average available for individuals with incomplete SCI to perform trunk movements (23), allows for fast real-time calculations, and fully covers the required leaning space. NNMF finds both the synergy matrix (*S*) and a coefficient matrix (*C*) to best approximate the activation store matrix (*A*).

The coefficient matrix along with $M_{\mathrm{store}}$ approximates the basis set (*B*) that defines the moments each synergy can produce at maximum activation. We found the basis set by solving

(8) $$M_{\mathrm{store},[2\times n]}=B_{\left[2\times 4\right]}C_{\left[4\times n\right]}$$

with a least-squares method that minimized the error of the norm. The basis set helps visualize the synergies and enables determination of the synergy activations to stabilize a leaning posture.

### 2.5. Controller design and operation

**Figure 3:**
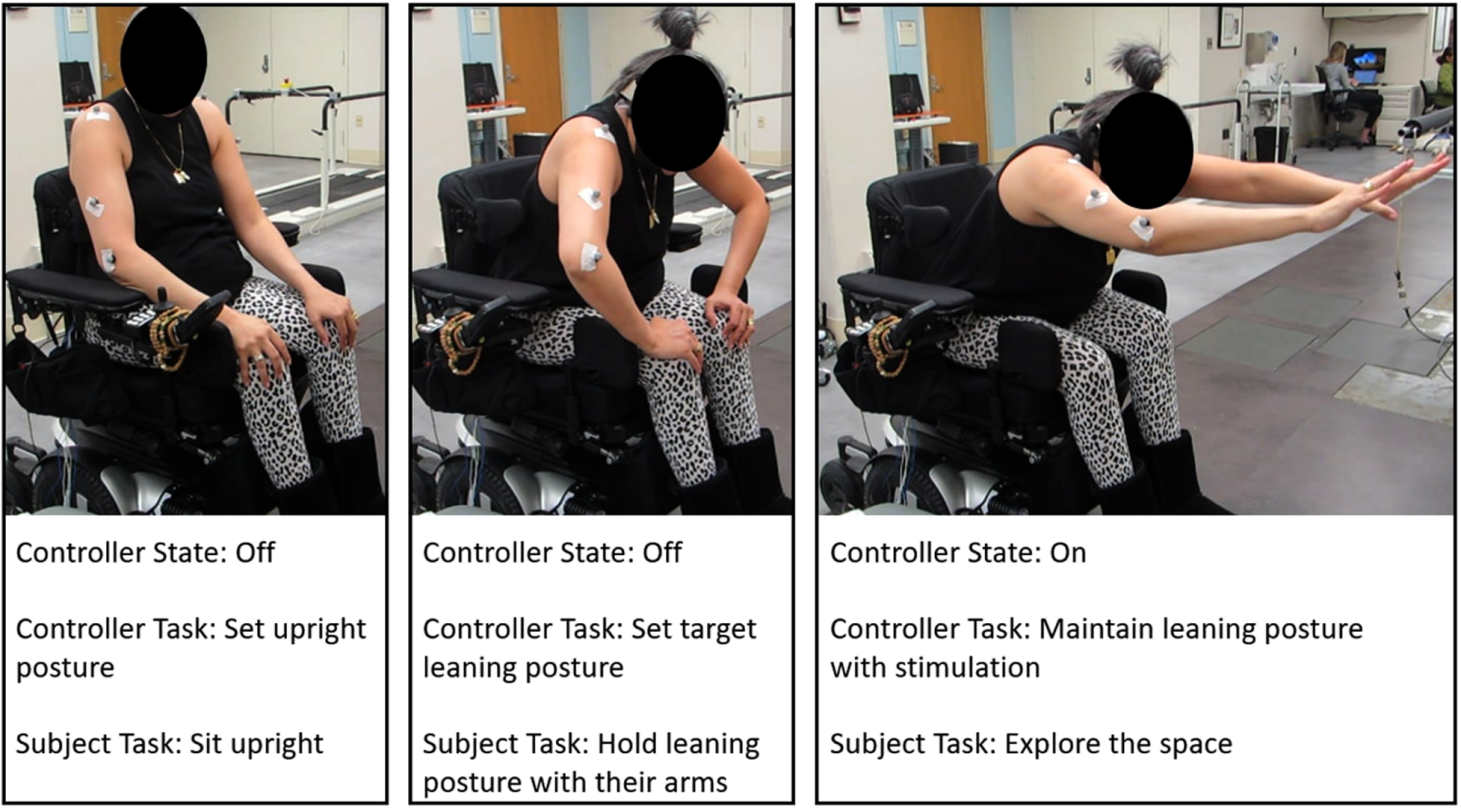
Operation of the controller. An upright posture is initially set, the subject then leans to a target posture and the controller is activated.

We obtained the feedback signal from an accelerometer (Trigno Avanti, Delsys Inc., Natick, MA) to estimate trunk tilt (pitch and roll angles), which was affixed to the back at the T4 level with double sided tape. Figure 3 shows the operation steps of the controller. Initially, the subject was asked to sit in an upright posture in their own wheelchairs, and the baseline pitch and roll angles were determined and subtracted from the measurements obtained during leaning to non-erect target postures. Subjects assumed leaning postures voluntarily through the actions of their upper extremities. The assumed pitch and roll angles were designated as the controller setpoints. The controller was then activated, and stimulation was ramped up over the course of one second to avoid causing large, rapid movements from a sudden change in stimulation. At this point the subject could raise their arms and rely on the controller to maintain the trunk at the setpoint. The controller operated at 40 Hz. At each sampling interval the posture error was obtained by subtracting the setpoint trunk angles from the current trunk angles (Figure 1). Error was routed to two PID controllers, one for each degree of freedom. PID controller outputs were added to the feedforward command. The feedforward command was the target moments required to support the subject at the setpoint posture and were obtained from the plane fits (∏(θ,ϕ)) defined in section 2.3.3. Once the feedforward moments were updated by the feedback values the resulting target moments were converted to synergy activations by solving

(9) $$M_{\mathrm{con},[2\times 1]}=B_{\left[2\times 4\right]}u_{\left[4x1\right]}$$

through a non-negative least squares optimization. Where $M_{\mathrm{con}}$ is the controller moments determined from adding the feedforward and feedback moments, *B* is the basis set determined in Equation 8, and *u* comprises the synergy activations. Synergy activations were converted to activation coefficients with

(10) $$\alpha_{\left[k\times 1\right]}=S_{\left[k\times 4\right]}u_{\left[4\times 1\right]}$$

and activation coefficients were then converted to stimulation PW by inverting the recruitment curves obtained via the methods in section 2.3.2 and applied to the appropriate channels of the neuroprosthesis.

#### 2.5.1. Tuning parameters

We incorporated some parameters into the control system that allowed for custom adjustments during the experiments for each subject. The proportional, integral, and derivative gains of the PID controller were respectively set to 0.5, 0.125, and 0.0125 Nm/deg based on preliminary tuning and did not require adjustment during the experiments. Channels that did not result in significant moment contribution during system identification were set to a percentage of their maximum comfortable level of stimulation. Despite showing little effect on the moment produced across the lumbar spine, these channels could still aid in stiffening the hip joints. Stimulation PW on these channels was initially set to 20% and increased to the highest value in which no visible lumbar motion occurred. This ensured stimulation levels were enough to anchor the pelvis while not causing lumbar motion. The feedforward command had scaling factors on both the desired pitch and roll moments. These were in place because the system identification and the controller experiments were performed in different seating conditions (trunk moment transducer vs. wheelchair), resulting in some inherent system identification errors. Additionally, a setpoint offset was available for cases where the initial stimulation caused the trunk to extend beyond the setpoint. In response, the PID controller decreases stimulation to levels that would prevent subjects from removing their arms from a support surface (wheelrims, armrests or thighs) without falling forward. The setpoint offset accounts for this initial trunk extension such that the resulting stimulation values enabled subjects to maintain the leaning postures without arm support.

### 2.6. Experimental implementation and outcome measures

Kinematics of the trunk and arm were obtained with a 16-camera motion capture system sampling at 100 Hz (Vicon Motion Systems Ltd., Oxford, UK). Reflective markers were applied to the C7 vertebrae, sacrum, and bilaterally on the acromion of the scapula, the anterior superior iliac spine of the pelvis, middle of the upper arm, the lateral and medial epicondyle of the elbow, and the middle metacarpophalangeal joint.

Each subject reached as far as possible in the forward, lateral, and downward directions. We examined performance under three conditions: reaching without stimulation, reaching with a constant level of stimulation, and reaching while the controller was active. Constant stimulation values were iteratively determined to provide a stable upright posture and are shown in Table 2.

**Figure 4:**
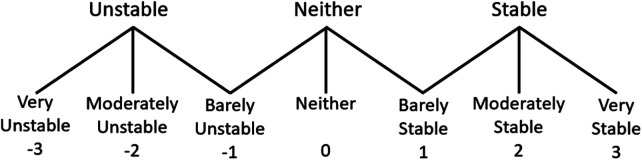
7-point Likert scale to access the subject's perceived stability during reaching.

For both the no stimulation and constant stimulation conditions we asked the subjects to reach as far forward, downward, left, and right as possible with their dominant hand. When reaching to their non-dominate side they were asked to use their non-dominant arm. Reaches in each direction were repeated three times both with constant stimulation and without stimulation. The degree of leaning was defined as the trunk pitch and roll angles at the point of maximum reach and were determined from motion capture data. These measures differ from the tilt information derived from the mounted accelerometer for real-time control. The trunk pitch and roll angles were defined as the angle between the global reference frame and the line defined between the sacrum and C7 marker (33). Maximum reaching distance was defined as the maximum distance in the reaching direction from the subjects C7 marker while sitting upright to the middle metacarpophalangeal joint while reaching. After each reach, the subjects were asked “How stable did you feel while reaching?” and shown a 7-point Likert-type scale (Figure 4), a modified version of the Usability Rating Scale (10, 34). Choices ranged from very unstable (−3) to very stable (3). Each reaching direction was repeated three times per condition.

Target postures were 30° pitch at −10°, 0°, 10° roll. Subjects assumed upright posture while the pitch and roll angles from the accelerometer were zeroed. Once at the target posture the controller was activated and the subject was asked to slowly stop supporting their trunk with their upper extremities. Negative roll angles indicate lateral leaning to the left. Once in a stable leaning posture we asked the subjects to reach as far as possible in the direction of leaning. For example, if the leaning posture was directly forward, we asked them to reach forward and downward. If the leaning posture was forward and to the left, we asked them to reach as far to the left as possible. The same outcome measures from the no and constant stimulation cases were determined from the leaning postures. Each leaning posture (forward, forward right, forward left) and reach direction (forward, down, left, right) was repeated three times with the leaning controller active.

At each posture, perturbations were applied manually at gradually increasing forces with a handheld loadcell attached to a padded surface recording forces at 100 Hz. While leaning directly forward, perturbations were applied to the back at the T4 level to induce forward trunk flexion. While leaning forward and laterally, perturbations were applied to the shoulder at the T4 level in the direction of leaning to induce increased lateral bending. The largest perturbation that each subject could resist without losing balance was isolated and the peak force and impulse were determined. Additionally, these were converted to moments about the lumbar joint by multiplying by the moment arm measured during the experiment as the distance between T4 and S1. The last outcome measure was the length of time a subject could hold a forward leaning posture. The subjects held a leaning posture of 30° pitch and 0° roll until the muscles were unable to maintain the posture. Length of time leaning was defined as the time from initiation of the controller to when the subject needed external support to remain upright.

### 2.7. Statistical analysis

For the maximum reach distance and degree of leaning outcome measures, the no stimulation, constant stimulation, and leaning controller conditions were tested for normalcy with an Anderson-Darling test and compared with a One-Way ANOVA. If significance was found a *post hoc* Tukey test was performed. The non-parametric survey data were analyzed with a Kruskal–Wallis test. If significant differences were found between conditions, multiple comparisons were performed with a Dunn's test. All statistical comparisons were performed with a single-subject experimental design where each subject served as their own control.

## 3. Results

### 3.1. System identification and synergies

**Figure 5:**
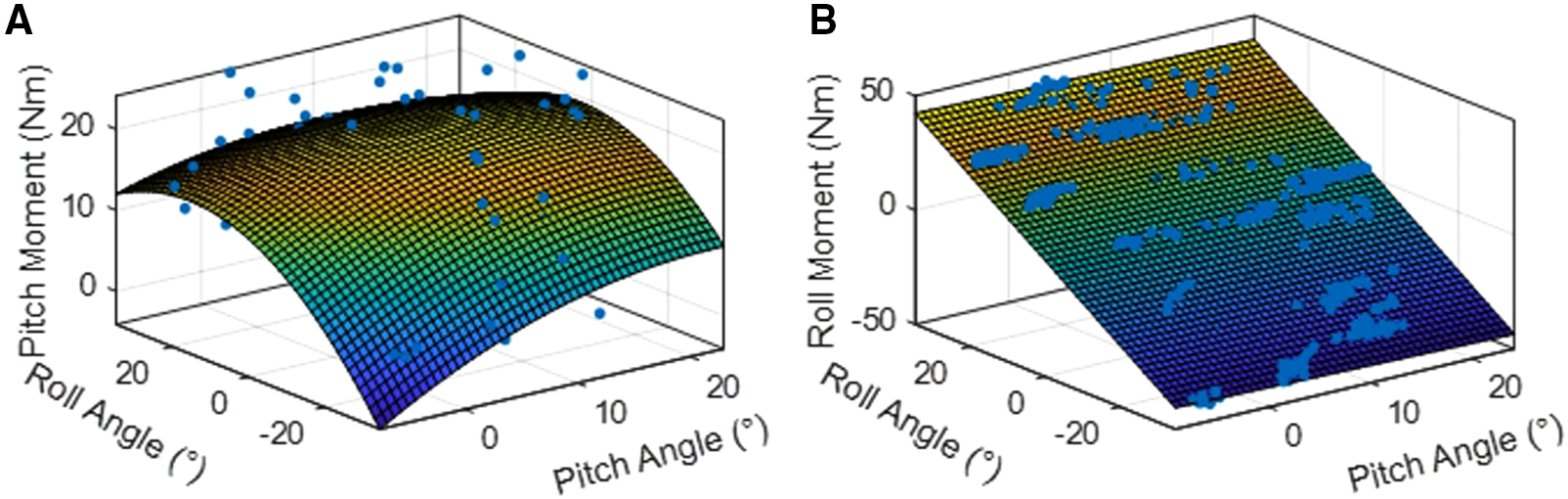
(**A**) second order polynomial surface fit of pitch moments produced from applying stimulation through the left erector spinae channel of subject S2. (**B**) Plane surface fit of the roll moments required to hold subject S2 in various leaning postures.

Figure 5A shows an example second order polynomial surface fit for the pitch moments produced from applying stimulating current through the left erector spinae channel and Figure 5B shows the plane fit for the required roll moments to support a leaning posture derived for subject S2. The RMSE and *r*^2^ values from the surface fits are shown in Table 3. The RMSE ranged from 0.21 to 9.95 Nm and the *r*^2^ values were greatest for the static postures and overall ranged from 0.06 to 0.97.

**Table 3:** Root mean squared error and coefficients of determination of the surface fits for the moments produced by each stimulus channel and the required moments to hold leaning postures.

	Root mean squared error (Nm)						Coefficient of determination (*r*^2^)					
	S1		S2		S3		S1		S2		S3	
	*M_p_*	*M_r_*	*M_p_*	*M_r_*	*M_p_*	*M_r_*	*M_p_*	*M_r_*	*M_p_*	*M_r_*	*M_p_*	*M_r_*
Static postures with no stimulation	7.68	5.07	5.56	4.55	9.95	9.8	0.91	0.95	0.90	0.97	0.35	0.87
Channel name												
Right erector spinae	2.65	0.83	3.94	1.73	2.61	1.23	0.36	0.58	0.35	0.66	0.12	0.48
Right surface erector spinae	2.54	0.9	2.6	0.95			0.77	0.64	0.48	0.74		
Right quadratus lumborum	3.62	1.02			4.07	2.55	0.28	0.61			0.35	0.49
Right surface quadratus lumborum	2.02	0.99	2.76	2.22			0.71	0.85	0.69	0.86		
Right iliopsoas			2.71	1.15					0.20	0.54		
Right semimembranosus	2.05	0.51			2.7	0.56	0.20	0.56			0.87	0.71
Right posterior adductor magnus					4.33	1.52					0.19	0.60
Right gluteus maximus					3.15	1.12					0.23	0.07
Right gluteus maximus 2					6.27	2.59					0.53	0.61
Left erector spinae	1.94	0.5	3.86	1.41	3	1.29	0.46	0.79	0.68	0.73	0.04	0.36
Left surface erector spinae	2.44	0.84	3.97	1.27			0.79	0.83	0.75	0.76		
Left quadratus lumborum	4.39	1.26	3.64	1.36			0.39	0.80	0.73	0.77		
Left surface quadratus lumborum	2.59	0.98	3.1	1.14			0.43	0.81	0.81	0.95		
Left iliopsoas			3.47	1.44					0.72	0.79		
Left semimembranosus					0.94	0.21					0.67	0.97
Left gluteus maximus	1.29	0.56					0.06	0.65				
Left gluteus medius	1.96	0.84					0.09	0.62				

**Figure 6:**
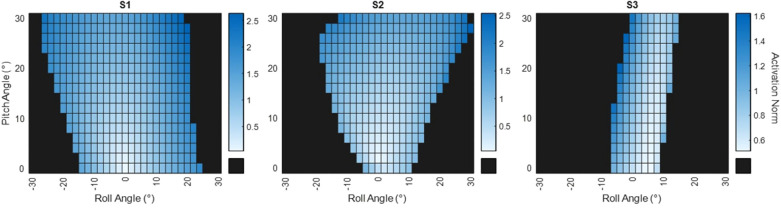
Heatmaps of the predicted leaning postures for each subject. Blue sections indicate leaning postures the subjects would be able to achieve. Darker blue indicates a higher activation vector norm showing greater amounts of stimulation would be required to hold that posture. Black areas show the subject would be unable to lean to those postures.

The results of the optimizations described in section 2.4 to determine the synergy calculations are shown in Figure 6. The heatmaps show the predicted range of leaning postures each subject could hold with stimulation. “Activation norm” refers to the norm of the activation coefficient vector (α) from Equation 2. Thus, darker blue areas indicate greater levels of activation. S1 exhibited the largest range of available postures from 20°/−20° roll to 30° pitch. S2 has reduced lateral leaning at postures closer to erect, however they were predicted to be able to hold lateral leaning postures while also leaning forward. S3 had the narrowest range of predicted leaning postures, ranging between −10° and 10° roll with up to 30° pitch.

**Figure 7:**
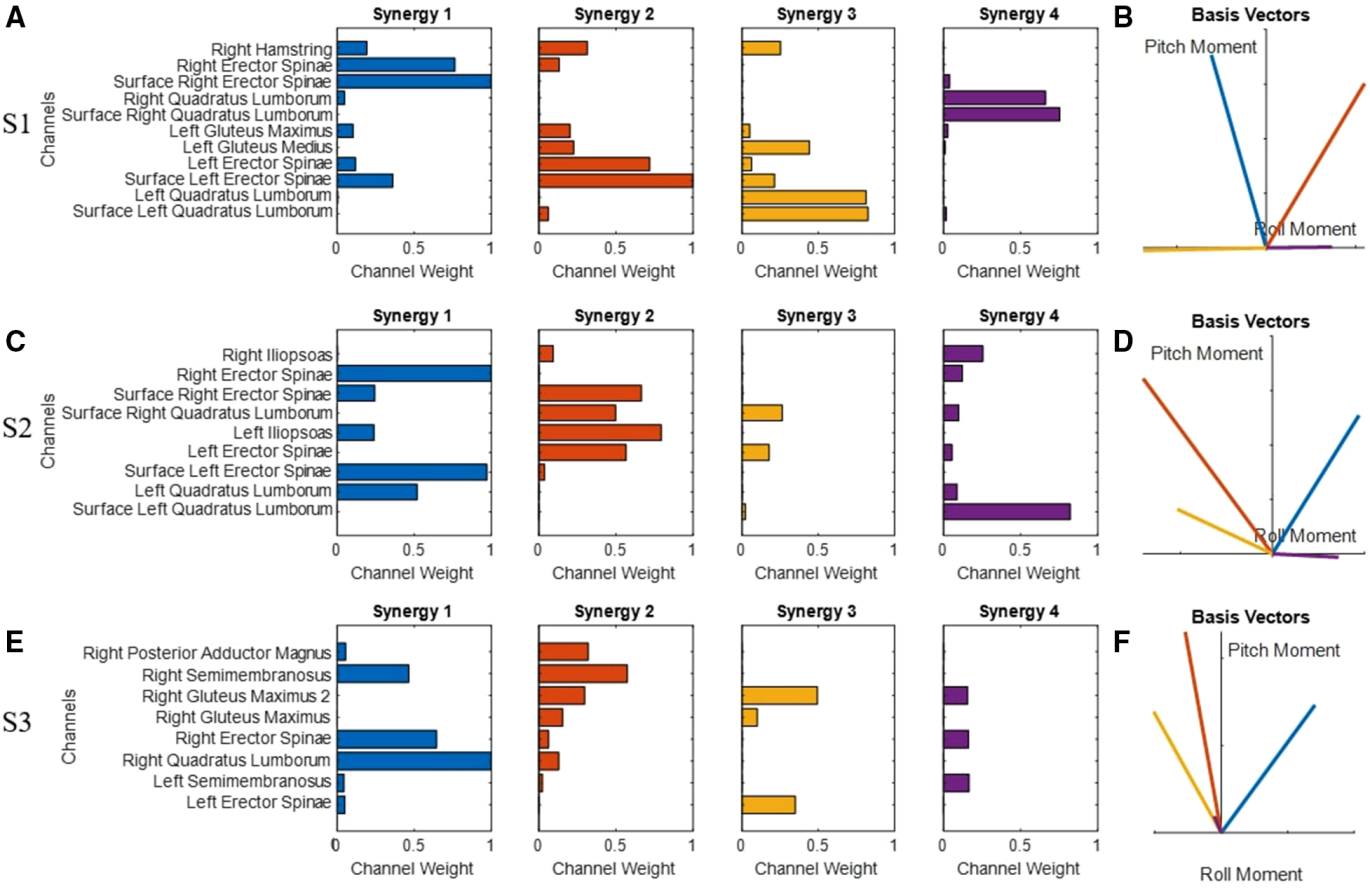
Identified synergies for subjects S1 (**A**), S2 (**C**), and S3 (**E**) and their respective basis sets (**B**,**D**,**F**). Synergies on the left correspond to the basis vectors on the right of the same color. Positive pitch moments indicate moments that would produce extension. Positive roll moments indicate moments that would produce right lateral motions.

The synergies (*S*) and basis set (*B*) for each subject are shown in Figure 7. Subject S1's synergy 1 and 2 resulted in basis vectors that primarily provided extension forces with opposing lateral biases. This was mostly accomplished through activation of the erector spinae with both implant and surface channels. Synergy 3 and 4 resulted in basis vectors that are directly lateral to the left and right through activation of the quadratus lumborum. Synergies 1 and 2 from Subject S2 resulted in basis vectors that produced moments in extension and lateral bending through activation of both the erector spinae and quadratus lumborum. Similarly, synergies 3 and 4 provided basis vectors that produced more lateral moments. S3 had synergies 2, 3, and 4 that produced lumbar moments to the left laterally and in extension. Synergy 1 produced a basis vector that results in extension and right lateral moments.

### 3.2. Experimental outcomes

**Figure 8:**
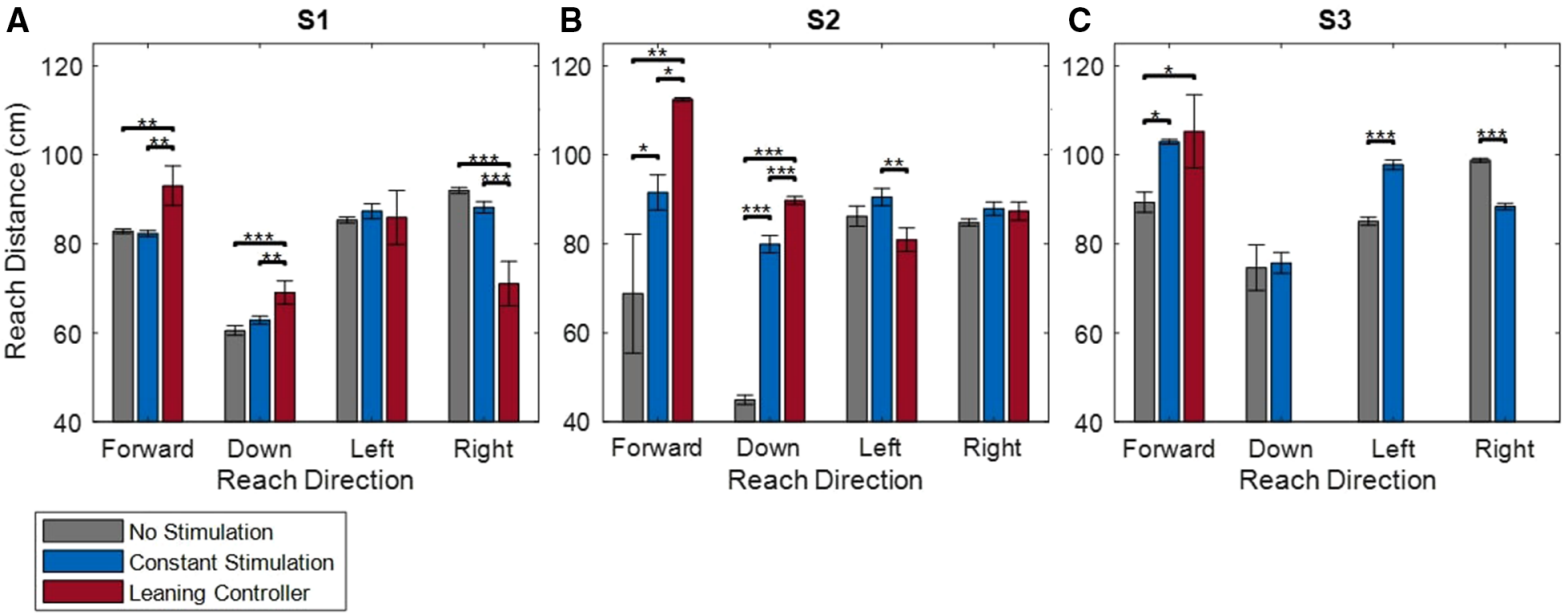
Reach distances for subjects S1 (**A**), S2 (**B**), and S3 (**C**) in the forward, down, left, and right directions. *indicates *p* < 0.05, **indicates *p* < 0.01, ***indicates *p* < 0.001.

Supplementary Video 1 shows the controller in action with a subject. The reaching distances for each subject under the three conditions (no stimulation, constant stimulation, and leaning controller) are shown in Figure 8. Forward reaching distances were increased by 10.2, 46.7, and 16 cm compared to no stimulation for Subjects S1, S2, and S3, respectively. Compared to constant stimulation, forward reaches increased by 10.7 and 20.9 cm for S1 and S2. No significant increase was observed for Subject S3. Leaning postures for Subject S3 were unable to support downward or lateral reaches, however the subject was able to raise their elbow in the target direction while leaning. The controller was unable to support farther arm movement. Subject S1 and S2 saw an increase of 8.6 and 44.9 cm respectively when reaching downward while leaning with the controller compared to without. The difference reduced to 6.2 and 9.9 cm respectively when compared to the constant stimulation. Lateral reaches either did not significantly improve with the leaning controller or resulted in decreased reaching distances.

**Figure 9:**
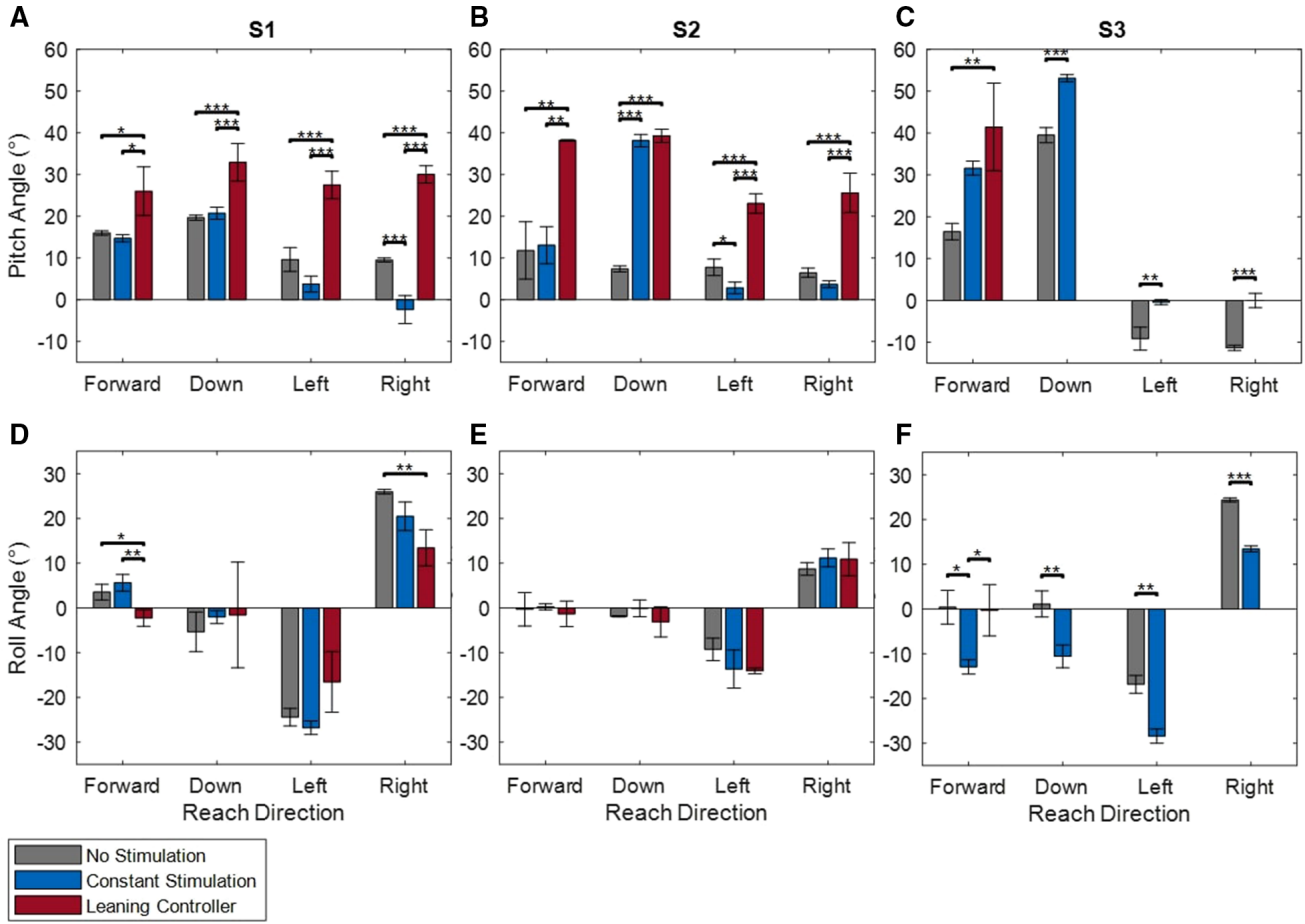
Pitch (**A**–**C**) and roll (**D**–**F**) angles for subjects S1 (**A**,**D**), S2 (**B**,**E**), and S3 (**C**,**F**) at the point of maximum reach in the forward, down, left, and right directions. *indicates *p* < 0.05, **indicates *p* < 0.01, ***indicates *p* < 0.001.

The pitch and roll angles of each subject during the moment of maximum reach distance are shown in Figure 9. Pitch angles while the leaning controller was active ranged from 25 to 40° were significantly increased over the no stimulation condition for all subjects and all directions where lateral leaning postures could be achieved (i.e., excluding subject S3). The leaning controller did not show a significant increase over constant stimulation while Subject S2 and S3 were reaching down and forward, respectively. During lateral leaning the controller supported roll angles of 10–20°, less than or equal to the angles the subjects could achieve without stimulation.

**Figure 10:**
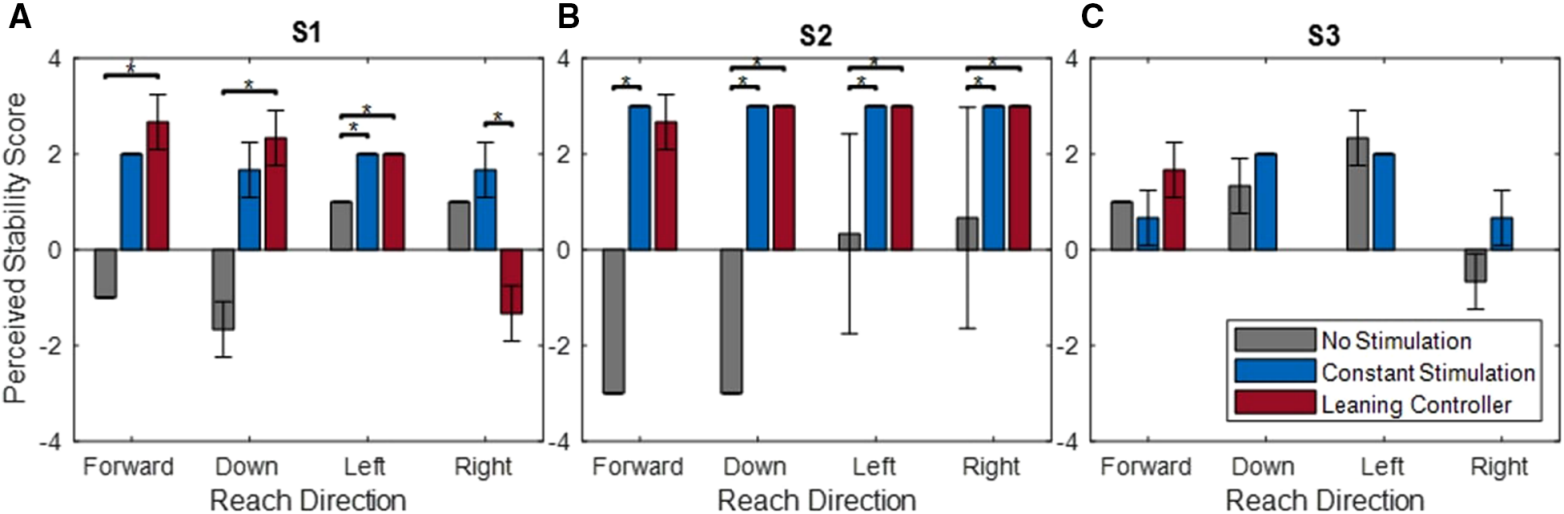
Perceived stability scores of subjects S1 (**A**), S2 (**B**), and S3 (**C**) during reaching tasks. Scores ranged from very unstable (−3) to very stable (+3). *indicates *p* < 0.05.

When the controller was activated, subjects rated their perceived stability during each reaching task as moderately stable or very stable (Figure 10). With the exception of S1 reaching to the right, these were in line with the perceived stability while constant stimulation was applied and were significantly higher than perceived stability while no stimulation was applied for Subjects S1 and S2. Without stimulation, Subjects S1 and S2 rated forward and downward reaches at barely to very unstable and lateral reaches as barely stable. Subject S3 had higher overall perceived stability with ratings typically at or above barely stable with no significant differences observed.

**Figure 11:**
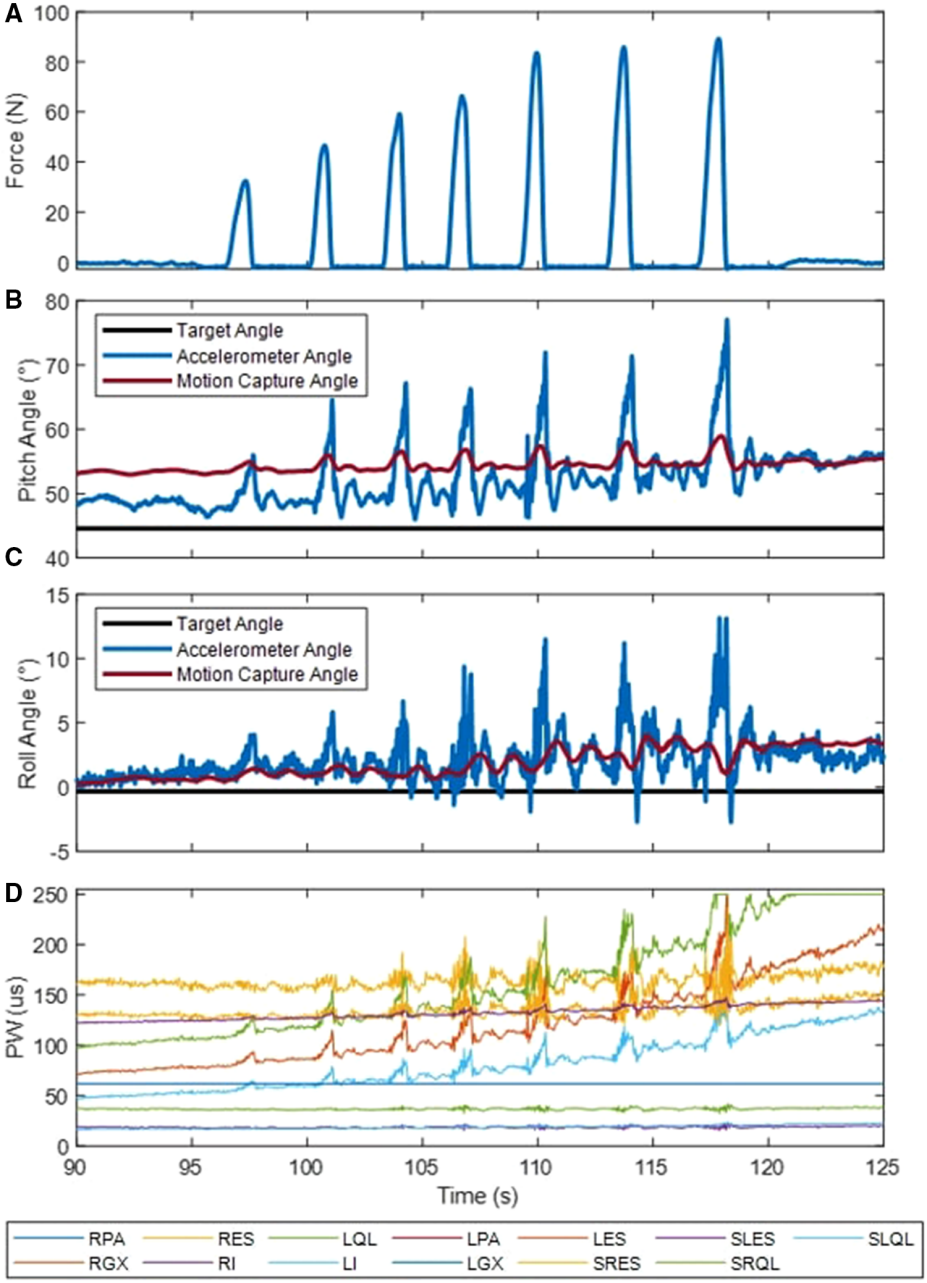
Example external perturbations applied to the back at the T4 level of S2. Perturbation forces are shown in section (**A**) Trunk pitch (**B**) and roll (**C**) angles determined via the body-worn accelerometer and the motion capture are shown along with the controller's setpoint. Stimulation commands (**D**) sent to the neuroprosthesis. Acronyms: R/l, Left/Right; ES, lumbar erector spinae; QL, quadratus lumborum; PA, posterior portion of adductor magnus; GX, gluteus maximus; GM, gluteus medius; IL, iliopsoas; S, surface stimulation.

Figure 11 shows an example perturbation trial while Subject S2 was leaning forward. In response to perturbations applied to the back at increasing levels, stimulation parameters changed to arrest the trunk motions away from the setpoint and return the subject back to the target posture. Table 4 quantifies the applied external perturbations; subjects S1, S2, and S3 were able to withstand forces of up to 32.5, 92.3, and 75.9 N, respectively. Subject S1 could respond to roughly the same level of perturbation to the back while leaning forward, and the right shoulder while leaning forward and to the left (29.2 and 32.5 N). Resistance to perturbation was lowest when applied to their left shoulder while leaning right (13.3 N). Subject S2 could resist large perturbations when applied to the back (92.3 N), however notably less force when applied to the right and left shoulder (19.2 and 16.9 N). Subject S2 had the greatest resistance to perturbation when force was applied to their left shoulder (75.9 N) and lower values on the back and left shoulder (20.6 and 31.6 N). Maximum moments and impulses follow the same trend as the maximum forces except for in Subject S1 where perturbations applied to the back and right shoulder were prolonged resulting in higher impulses. These results demonstrate that the controller can accommodate externally applied perturbations.

**Table 4:** The maximum perturbation forces, moments, and impulses each subject was able to resist while leaning under the influence of the controller.

Subject	Leaning direction	Perturbation location	Maximum force (N)	Maximum force (% Bodyweight)	Maximum moment (Nm)	Maximum impulse (Ns)
S1	Forward	Back	29.2	5.1	10.4	45.4
	Forward left	Right shoulder	32.5	5.7	11.5	149.5
	Forward right	Left shoulder	13.3	2.3	4.7	5.1
S2	Forward	Back	92.3	11.9	42.5	61.1
	Forward left	Right shoulder	19.2	2.5	8.8	10.0
	Forward right	Left shoulder	16.9	2.2	7.8	12.3
S3	Forward	Back	20.6	2.3	13.6	9.1
	Forward left	Right shoulder	31.6	3.5	20.8	19.7
	Forward right	Left shoulder	75.9	8.5	50.2	36.9

**Figure 12:**
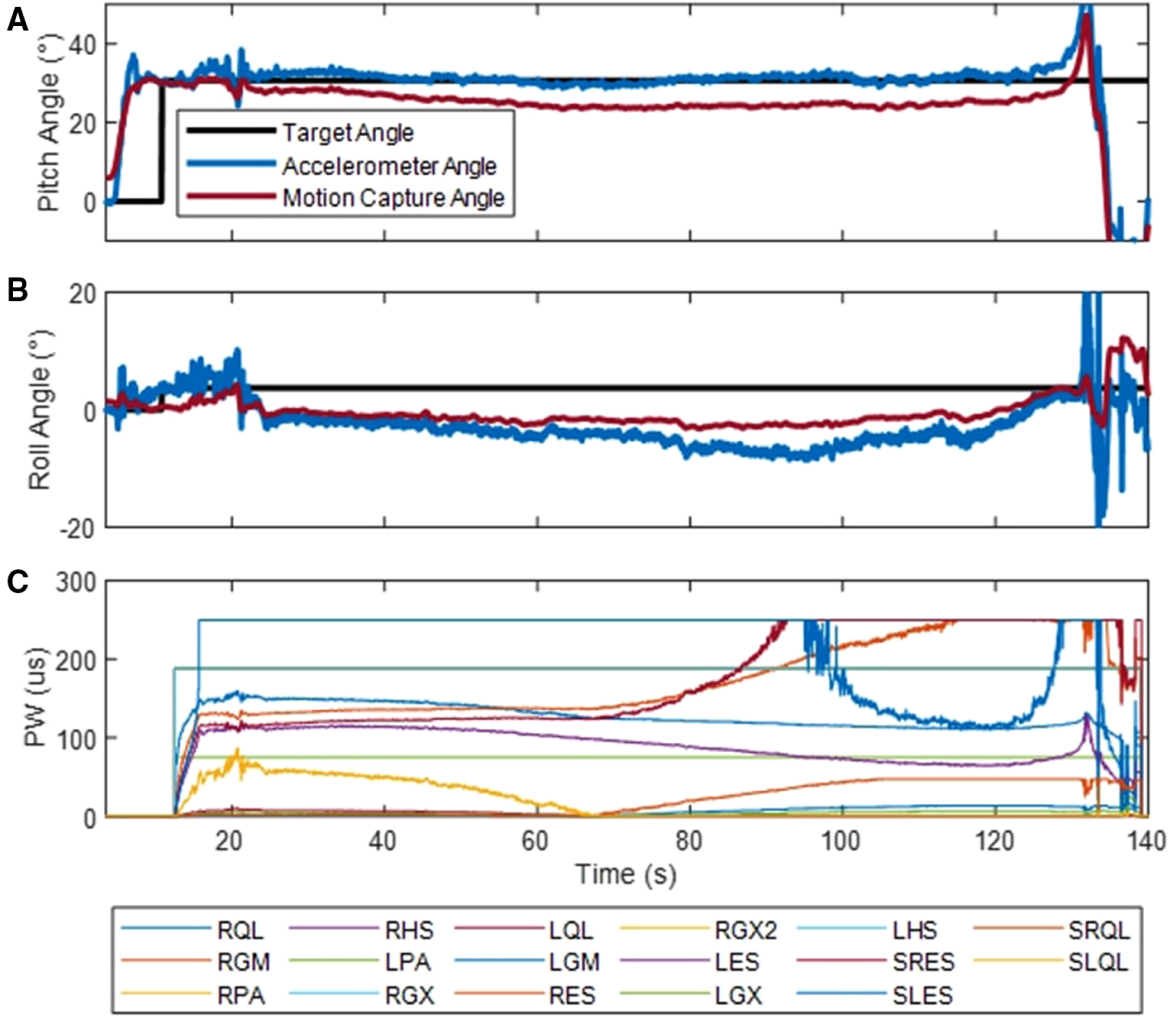
Example trial determining how long S1 could maintain a leaning posture. Trunk pitch angle (**A**) and trunk roll angle (**B**) were determined through tilt of the accelerometer and the motion capture system. The target angle represents the controller setpoint. Stimulation pulse width (PW) are shown in (**C**) Acronyms: R/l, Left/Right; ES, lumbar erector spinae; QL, quadratus lumborum; PA, posterior portion of adductor magnus; GX, gluteus maximus; GM, gluteus medius; IL, iliopsoas; S, surface stimulation.

An example trial to determine how long Subject S1 could hold a leaning posture is shown in Figure 12. The controller was engaged at 12 s and maintained a leaning posture at roughly 30° trunk pitch for 117 s until the muscles fatigued and the subject began to flex forward away from the setpoint. While the controller maintained the pitch setpoint angle during this time (Figure 12A), the initial application of stimulation resulted in an offset between the roll setpoint angle and the accelerometer tilt measure (Figure 12B). Stimulation pulse widths were modified throughout the trial to both maintain trunk pitch and correct the trunk roll. Subjects S1, S2, and S3 were able to maintain seated postures for 117, 112, and 45 s, respectively.

## 4. Discussion

We designed and deployed stimulation feedback controllers that enabled individuals with SCI to attain and maintain unsupported leaning postures previously unobtainable by their own volition without self-assisting with both arms. From our review of the literature, stable static leaning postures with neural stimulation have not previously been reported in individuals with motor complete SCI. Other recent advancements have focused primarily on supporting upright posture by resisting both externally (11, 12) and internally applied (13) perturbations. Additionally, prior reports describe controllers designed to return a user upright after exceeding a leaning threshold, rather than maintain a non-erect setpoint (35, 36). Our controller enabled leaning postures that increased reaching distances by up to 46 cm, thus expanding available workspace and potentially aiding in activities of daily living. These non-upright postures were resistant to external perturbations, were generally perceived to be stable, and facilitated leaning for almost 2 min.

An important consideration of any assistive technology is its safety profile (37), but even the safest systems will be abandoned if they do not instill a sense of trust and security in their users (38). We investigated how secure our subjects felt during reaches by quantifying their perceived stability. The constant stimulation pattern served as a positive control as it has been shown to maintain upright posture (10). As expected, the subjects perceived the constant stimulation condition as stable while sitting erect. While leaning, the subjects also rated their perceived stability as moderately to very stable. This was an improvement over reaching with no stimulation, which was generally considered unstable. Subject S3 rated all but one of the conditions as stable, restricting any potential for improvement with stimulation. Overall individuals felt stable while leaning in typically unstable postures with our controller design.

The mapping of muscle moments with the polynomial surface fits showed similar efficacy to polynomial fits in the past (16). Alternative methods such as neural networks (39) or Gaussian process regressions (15) might offer improvements but can be prone to overfitting especially with small data sets (40). The plane fits of the required moments to hold leaning postures resulted in *r*^2^ values above 0.9 for subject S1 and S2. Suggesting that the required support moments are approximately linear up to 30°.

The ability to resist perturbations is an important requirement in cases where the individual is bumped during a leaning action or is interacting with the environment. Perturbations were not intended to assess the extreme limit of forces the subjects could resist. Instead, it was intended to demonstrate the ability of the controllers to accommodate perturbations of varying sizes without losing stability. The controller presented was capable of resisting perturbations of up to 92 N and 12% bodyweight for Subject S2. These magnitudes of applied disturbances were significantly lower than the 45% bodyweight perturbations individuals were able to resist with an upright controller (12). However, a significant amount of muscle force is required to hold the leaning postures with typically only a small amount of additional muscle recruitment available to respond to perturbations. To put these values into perspective, summing the moments about the lumbar joints while the users leaned 30° forward suggests that S1, S2, and S3 could hold a weight of 6.2, 17.8, and 3.2 kg at an outstretched arm length of 30 cm, respectively. These values are slightly lower than those predicted for the upright posture by Lambrecht et al. (41) and Friederich et al. (27).

FNS is often limited by the contractile strength of the paralyzed muscles and the number of muscle fibers available for recruitment. Maximum isometric force of paralyzed muscles is estimated to be 30%–75% of able bodied (42, 43) and neuroprostheses can only activate a limited number of muscle groups with the available stimulation channels. Forward leaning was successful in all subjects because the majority of channels available produced strong extension moments (Figures 7B,D,F), however lateral leaning postures were more difficult to sustain because of the reduced number of channels that produced moments in the medial-lateral direction. Additionally, the lack of trunk flexors prevented the controller from counteracting extension moments to produce purely lateral moments by muscles with multiple lines of action. The iliopsoas is intended to provide hip and lumbar spine flexion (41), however when stimulating Subject S2's iliopsoas channel, the output moment resulted in extension. The channel likely excited adjacent nerves activating the erector spinae innervated at the same spinal level. Such “spillover” is documented to occur with surface or intramuscular electrodes (44). This further emphasizes the need to characterize channels based on their functional outputs and not the intended target (27).

To achieve lateral leaning, subjects leaned forward 30° and laterally 10°, postures we predicted to be achievable for Subjects S1 and S2 (Figure 6). Subjects S1 and S2 attained these postures and successfully reached laterally with their arms while maintaining it. However, reach was not significantly extended due to the limited 10° of trunk lateral bending. Results shown in Figure 6 also predicted that subject S3 would have limited lateral capabilities compared to subjects S1 and S2, with the targeted posture being near the outer limits of feasible postures. These predictions were confirmed experimentally, when Subject S3 achieved these postures. However, when asked to reach laterally, they were only able to raise their elbow without full forearm extension confirming we were near the limits of his capabilities.

A possible avenue for improving lateral leaning is by incorporating stiffness into the controller. Increased stiffness is obtained through co-contraction of antagonist muscles and has been postulated to improve simulated FNS reaching by increasing perturbation resistance (45). Stimulation of antagonist trunk muscles has been reported to increase stiffness in able-bodied individuals (46). Increasing trunk stiffness may also improve subjective perception of stability. Stiffness could be introduced by modifying the synergies to include a baseline level of antagonistic muscle activation or by incorporating baseline co-contraction in the controller's real-time optimization step (Equation 9).

The synergy control scheme previously used to control the arm of able-bodied individuals (16) has now been proven to work in individuals with SCI for trunk control. While controversy exists about whether the nervous system controls movement through these synergies (47), it has been shown as a viable method of controlling multiple actuators over multiple degrees of freedom both in simulations (48) and able-bodied individuals (16). This report extends its applicability to individuals with paralysis. The synergy control scheme has an advantage of being based directly on the forces and moments produced by the individual and is computationally tractable enough for real-time control on a fully implanted system.

Home use of control systems such as those we have developed have the potential to extend the workspace of neuroprosthesis users thus aiding in activities of daily living and increasing independence. Yet, performance in the home and community settings and value to the system recipients remains to be quantified, and should be an active research priority for future studies. Anecdotally, one subject stated that the leaning action would be perfect for instances where an object is just out of reach, such as grabbing an item out of the cabinet while cooking.

### 4.1. Limitations

There are several limitations of the work presented here. The leaning controller was only tested with three individuals. All subjects achieved leaning postures otherwise unobtainable; and all achieved extended reaching capabilities. However, we do not claim the controller would be generalizable to the entire SCI population. Instead, the subject specific system identification utilized allows us to predict the obtainable leaning postures from the controller and determine which individuals could benefit. Second, despite the custom system identification the controller still required initial tuning before being able to support leaning postures. Commonly, the feedforward pitch moment was scaled down because otherwise stimulation caused subjects to return to an upright posture. The requirement for initial tuning could be because system identification was performed in a custom chair while the leaning experiments were performed in the subjects’ own wheelchairs, resulting in differences in seating posture. Future work should explore ways to improve the system identification to minimize, automate, or eliminate the need for tuning. Currently, our controller is only capable of holding a non-upright posture that the subject has already assumed. Future controllers should explore ways to deploy the subject's trunk to the target posture and return them to an upright posture after the desired task is completed. Finally, the neuromuscular system is nonlinear, time-varying, and time-delayed. Improvements to controller performance may be obtained by substituting the linear PID system with a nonlinear controller such as sliding mode (49) or model predictive control (50). As the objective of the current study was to maintain non-upright postures, the small movements around the target posture could be approximated as linear, thus allowing the computationally less expensive linear PID controllers to be better matched to the control task. More advanced control architectures may result in better outcomes; and this is a subject of future work.

## 5. Conclusion

Restoring the ability to maintain non-upright postures after paralysis by SCI is possible through application of feedback control of stimulation with imposed synergies of the trunk and hip muscles. This results in extended anterior and lateral reach, and has the potential to increase the functional capabilities of individuals with motor complete SCI while seated in their wheelchairs and facilitate many activities of daily living. In this study, we designed and implemented a synergy-based FNS controller capable of maintaining non-erect postures through electrical activation of typically paralyzed trunk and hip muscles. The leaning postures increased reach by up to 46 cm compared to no stimulation and resisted externally applied perturbations of up to 90 N. These postures were perceived to be stable by the users indicating that they were confident and comfortable while leaning with the neuroprosthesis. Implementation of leaning motions in neuroprostheses can expand available workspace and provide increased options for individuals with SCI.

## Data Availability

The raw data supporting the conclusions of this article will be made available by the authors, without undue reservation.
